# GPS Data and Machine Learning Tools, a Practical and Cost-Effective Combination for Estimating Light Vehicle Emissions

**DOI:** 10.3390/s24072304

**Published:** 2024-04-05

**Authors:** Néstor Diego Rivera-Campoverde, Blanca Arenas-Ramírez, José Luis Muñoz Sanz, Edisson Jiménez

**Affiliations:** 1 Machine-Engineering Division, Escuela Técnica Superior de Ingenieros Industriales—ETSII, Universidad Politécnica de Madrid—UPM, 28006 Madrid, Spain; joseluis.munozs@upm.es; 2 Grupo de Investigación en Ingeniería del Transporte, Universidad Politécnica Salesiana, Cuenca 010105, Ecuador; ejimenezl2@est.ups.edu.ec; 3 Instituto Universitario de Investigación del Automóvil Francisco Aparicio Izquierdo—INSIA-UPM, Escuela Técnica Superior de Ingenieros Industriales—ETSII, Universidad Politécnica de Madrid—UPM, 28006 Madrid, Spain; blanca.arenas@upm.es

**Keywords:** low-cost emission model, machine learning model, portable emissions measurement system, emission parametric model, real driving emissions

## Abstract

This paper focuses on the emissions of the three most sold categories of light vehicles: sedans, SUVs, and pickups. The research is carried out through an innovative methodology based on GPS and machine learning in real driving conditions. For this purpose, driving data from the three best-selling vehicles in Ecuador are acquired using a data logger with GPS included, and emissions are measured using a PEMS in six RDE tests with two standardized routes for each vehicle. The data obtained on Route 1 are used to estimate the gears used during driving using the K-means algorithm and classification trees. Then, the relative importance of driving variables is estimated using random forest techniques, followed by the training of ANNs to estimate CO_2_, CO, NO_X_, and HC. The data generated on Route 2 are used to validate the obtained ANNs. These models are fed with a dataset generated from 324, 300, and 316 km of random driving for each type of vehicle. The results of the model were compared with the IVE model and an OBD-based model, showing similar results without the need to mount the PEMS on the vehicles for long test drives. The generated model is robust to different traffic conditions as a result of its training and validation using a large amount of data obtained under completely random driving conditions.

## 1. Introduction

Vehicle emissions from internal combustion engines are the primary source of pollution in urban areas, negatively impacting air quality in cities [1]. Consequently, these pollutants need to be quantified [2]. Thus, vehicular emissions inventories serve as important tools for implementing and evaluating policies aimed at reducing the environmental impact of vehicular activity on the quality of life of the population [3]. The quality of emissions inventory results directly depends on the inputs and methodologies applied in their determination; therefore, various methods exist for estimating pollutants according to the realities of each population. Among the most commonly used alternatives are the International Vehicle Emissions (IVE) model developed in the United States by the Massachusetts Institute of Technology in collaboration with the International Council on Clean Transportation and the Computer Program to Calculate Emissions from Road Transport (COPERT) developed in the European Union by the Joint Research Center. These models estimate vehicular pollution emissions based on parameters such as emission factors, vehicular activity, and characteristics of the vehicle fleet. However, these parameters may not be equivalent to those in regions like Latin America, as variations in geographical and environmental conditions, vehicle technology, driving styles, and fuel quality can significantly impact vehicle emissions, as determined by [4], and may not be fully reflected in the IVE and COPERT calculations [5]. Therefore, different authors have developed methods to improve pollutant estimation by considering the specific conditions of each region or city. Costagliola et al. [6,7] found that pollutant emissions estimated using laboratory chassis dynamometer tests and adjusted driving cycles are lower than those determined in real driving cycles. Kurtyka et al. [8] and Mera et al. [9] reach similar conclusions, emphasizing that the differences in results between dynamometer tests and real driving emissions (RDEs) are due to traffic conditions and driving styles. Hence, they recommend evaluating pollutant emissions in real driving cycles.

Fontaras et al. [10] and Samaras et al. [11] determined that trips in private vehicles constitute the main cause of fuel waste and unnecessary emissions of pollutants, influenced by driver behavior, route selection, and traffic management, highlighting the importance of vehicle monitoring for large-scale pollutant estimation. Prakash and Bodisco [12] and Boulter et al. [13] determined that fuel consumption and pollutant emissions depend on vehicle-specific factors such as model, engine displacement, weight, fuel type, technological level, and mileage, as well as operational factors such as speed, acceleration, road gradient, ambient temperature, and especially the gear shifting strategy employed by the driver [14,15,16]. Rivera-Campoverde et al. [17] proposed a model based on machine learning and OBDs (on board diagnostics) for estimating emission factors of a single vehicle through real short-duration driving tests in Cuenca-Ecuador, thus avoiding long measurement campaigns and prolonged use of PEMSs (portable emissions measurement systems). Other authors, such as [18,19], proposed GPS-based models that consider real traffic conditions, obtaining good results with low implementation costs.

This article presents a novel method for estimating pollutant emissions from three different types of vehicles, using driving variables such as speed and gradient obtained through GPS, as well as characteristic parameters of each vehicle such as mass, engine displacement, and aerodynamic coefficients through the application of machine learning techniques. To achieve this, RDE tests were conducted on three routes, from which emissions, GPS, and OBD data were collected. With these data, the input variables of the model and their respective levels of importance were estimated, followed by the training of an artificial neural network (ANN) validated with data obtained from three different RDE tests not used for training, confirming the validity of the emissions estimator. Finally, this estimator was applied to a dataset of 324, 300, and 316 km of real driving data for each vehicle. The results were compared with those obtained from the IVE and OBD test models, showing similar outcomes.

## 2. Materials and Methods

### 2.1. Methodology for the Estimation of Emission Gases under Real Driving Conditions

Pollutant emissions must be measured under real driving conditions [20]. Within these results, various factors are considered, such as driving style, fuel type, geographic location, and environmental conditions in which vehicles are operated [11], which are currently not considered in the models used by the Mobility Company of the city of Cuenca (EMOV-EP).

**Figure 1:**
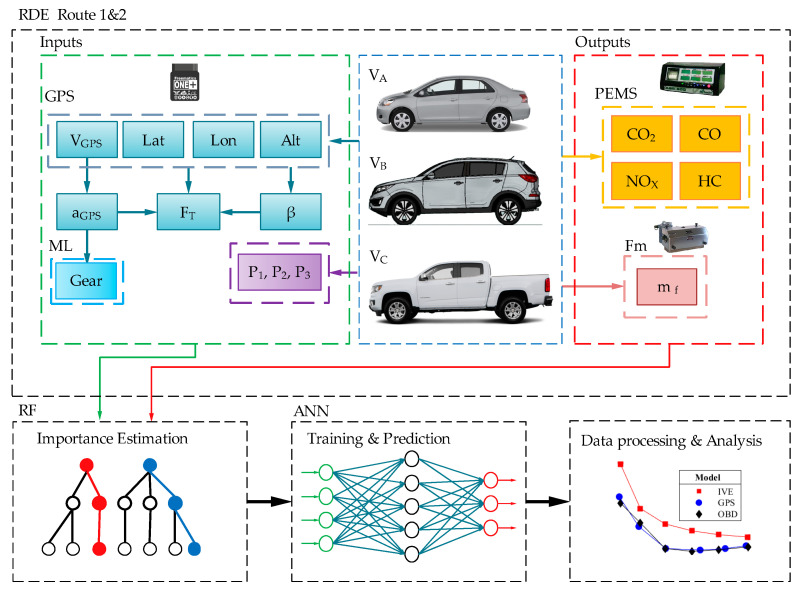
Methodology and proposed procedure.

To estimate pollutant emissions using a parametric model that considers the weight, engine displacement, and aerodynamic coefficients of the vehicle under real driving conditions, the following steps are proposed, as illustrated in Figure 1:

1. Acquisition of real driving and emission data on two routes based on [20] for each vehicle.
2. Estimation of the relative importance of each obtained variable.
3. Training and validation of the neural network with the most significant variables from route 1.
4. Validation of the trained ANNs using data from Route 2.
5. Application of the random driving dataset to the validated ANNs.
6. Processing and presentation of results.

For data collection, the vehicles used are the best-selling ones in Ecuador in the Sedan, SUV, and pickup categories. According to [21], the vehicles, whose characteristics are shown in Table 1, undergo all maintenance operations recommended by the manufacturer. Additionally, the aerodynamic characteristics of the vehicle are displayed, such as the drag coefficient (***C_X_***) and the frontal area of the vehicle (***A_f_***).

The portable emissions measurement system (PEMS) used is the Brain Bee AGS-688 gas analyzer, powered by a battery independent from the test vehicles, as established in [20]. Fuel consumption is measured using the AIC Fuel Flow Master 5004. The GPS used is incorporated within the Freematics ONE+ data logger, which stores latitude (***Lat***), longitude (***Lon***), altitude (***Alt***), and vehicle speed (***V_GPS_***) data on an SD card in CSV format. In addition to GPS data, the device stores driving data from OBD such as vehicle speed (***V_OBD_***). The obtained data are shown in Table 2.

### 2.2. Test Routes

**Figure 2:**
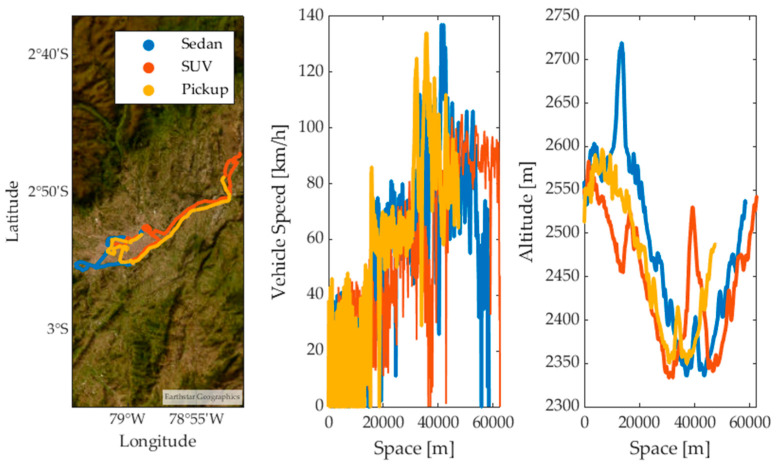
Test routes.

To analyze the behavior of the test vehicles during the application of the RDE tests [20], two different routes were proposed: Route 1 and Route 2. The datasets of each vehicle obtained on Route 1 were divided into 70% for training, 15% for validation, and the remaining 15% for testing the ANNs. The datasets of each vehicle obtained on Route 2 were used for a double cross-validation of the trained ANNs. The data collection routes used in the various RDE tests are located in the city of Cuenca, Ecuador. Urban segments are located in the city center, rural segments on the North Pan-American Highway, and highway segments on the Cuenca–Azogues highway, as shown in Figure 2.

The tests were conducted without the presence of rain or strong winds, with the windows closed and without air-conditioning activated. The test vehicles carried two passengers and a full tank of fuel. According to the manufacturer’s recommendations, 92-octane fuel was used. The characteristics of the routes in real driving conditions are shown in Table 3 and are validated according to the guidelines in [20].

### 2.3. Estimation of Pollutants

Based on the volumetric concentrations of pollutants in the exhaust gases measured by the PEMS, the mass flow rates of each pollutant were estimated using the procedure described in [20]. The exhaust mass flow rate ${\dot{m}}_{ex}\;[g/s]$ was estimated from the mass flow rate of air ${\dot{m}}_{in}$, which was estimated from parameters obtained from OBD, and the fuel flow ${\dot{m}}_{f}$, measured by the flow meter located in the fuel line.

(1) $${\dot{m}}_{ex}={\dot{m}}_{in}+{\dot{m}}_{f}$$

The emissions of pollutant *j* measured on a dry basis ${\;C}_{dry,j}$ were corrected to a wet basis $C_{wet,\;j}$ using the correction factor $k_{w}$, which depends on the molar ratio of hydrogen α and the concentrations of *CO_2_* and *CO* on a dry basis, $C_{{CO}_{2}}+C_{CO}$, respectively.

(2) $$C_{wet,\;j}=k_{w,j}{\;C}_{dry,j}$$

(3) $$k_{w}=\frac{1.008}{1+0.005\alpha {(C}_{{CO}_{2}}+C_{CO})}$$

**Figure 3:**
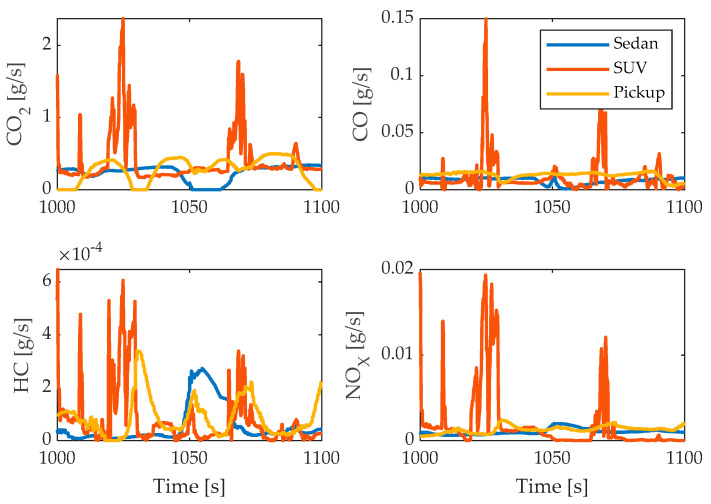
Tailpipe emissions of CO_2_, CO, HC, and NO_X_.

The instantaneous mass emissions of each pollutant ${\dot{m}}_{j,\;i}\;[g/s]$ are obtained from the instantaneous concentration of each gas $c_{j,}$ and the ratio between the density of each component and the overall density of the exhaust $\mu_{j\;}$. According to [20] the values of $\mu_{j}$ are as follows: $\mu_{{CO}_{2}}=0.001518,\;{\;\mu }_{CO}=0.000966,\mu_{HC}=0.000499,\mu_{{NO}_{X}}=0.001587$. The instantaneous emissions of pollutants obtained during real driving tests are shown in Figure 3.

(4) $${\dot{m}}_{j,\;i}=c_{j,\;i}\mu_{j,\;i}\;{\dot{m}}_{ex,i}{\;10}^{-3}$$

The emissions of each pollutant $m_{j}$ (g) in the driving cycle are equal to the sum of n elements of their instantaneous emissions over time for a sampling time ∆t equal to 0.1 s.

(5) $$m_{j}=\sum_{i=1}^{n}{\dot{m}}_{j,i}\;∆t$$

**Figure 4:**
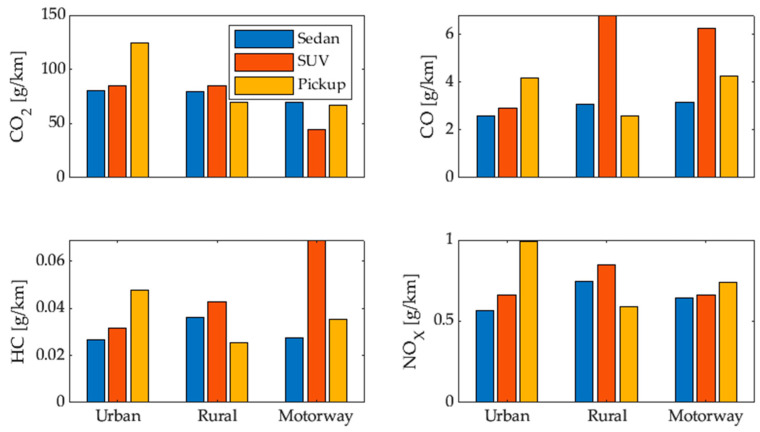
Emission factors of each vehicle per section of the RDE test.

The emission factors $EF_{j,k}$ of each pollutant ([g/km]) are determined by the following equation:

(6) $${EF}_{j,k}=\frac{m_{j,k}}{s_{k}}$$

where $m_{j,k}$ is the mass of pollutant *j* and s is the distance traveled in section *k* of the RDE test, where *k* takes the values of *u*, *r*, *m* for the urban, rural, and highway sections, respectively. The emission factors of each vehicle per section are shown in Figure 4.

Applying the total emissions generated for each pollutant and the total distance traveled during the RDE test to Equation (6) yields the average emission factors for each vehicle, which are shown in Table 4.

### 2.4. Predictor Estimation

Among the most influential variables in pollutant emissions, characteristics inherent to individual vehicles stand out, such as engine displacement. This is because larger engines burn more fuel per cycle, resulting in a greater generation of CO_2_, CO, HC, and NO_X_ [22]. It is important to consider that the specific influence of engine displacement on emissions may vary depending on the engine design, technology, and implemented emissions control.

Another variable analyzed in pollutant emissions is the vehicle’s weight [23], as it influences the rolling resistance force *F_r_*, which is shown in Equation (7), and depends on the coefficients of static adherence *f* = 0.015 and dynamic adherence *f*_0_ = 0.01, as well as affecting the gravitational resistance force *F_g_* shown in Equation (8).

(7) $$F_{r}=mg\left(f+f_{0}{V_{GPS\;i}}^{2.5}\right)$$

(8) $$F_{g}=mg\sin\left(\frac{{Alt}_{\;i+1}-{Alt}_{\;i}}{S_{i+1}-S_{i}}\right)$$

The aerodynamic resistance *F_a_* is one of the major contributors to the fuel consumption and pollutant emissions of a vehicle, especially when traveling at high speeds [24]. It is calculated using Equation (9) [25], where the value of air density ρ is equal to 0.89 kg/m³.

(9) $$F_{a}=\frac{1}{2}\rho {{C_{X}A_{f}V}_{GPS\;i}}^{2}$$

**Figure 5:**
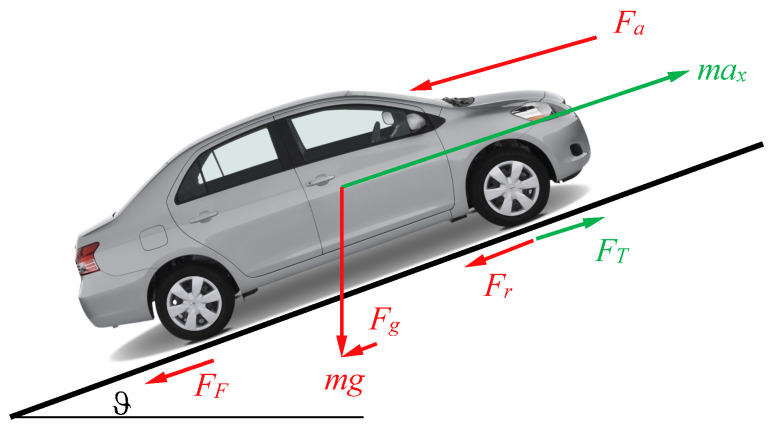
Active forces during circulation.

The longitudinal acceleration of the vehicle is determined by Equation (10), while the forces occurring during driving are applied as shown in Figure 5 and are related using Equation (11), where *F_T_* represents the tractive force and *F_F_* represents the braking force, and they are mutually exclusive.

(10) $$a_{x\;i}=\frac{V_{GPS\;i+1}-V_{GPS\;i}}{t_{i+1}-t_{i}}$$

(11) $$F_{T}-F_{F}=ma_{x}+F_{r}+F_{g}+F_{a}$$

For the training of machine learning architectures, parameters *P*_1_, *P*_2_, and *P*_3_ are considered, which refer to the engine displacement, vehicle weight, and its aerodynamic characteristics (*C_X_*, *A_f_*), respectively.

### 2.5. Estimation of the Selected Gear

**Figure 6:**
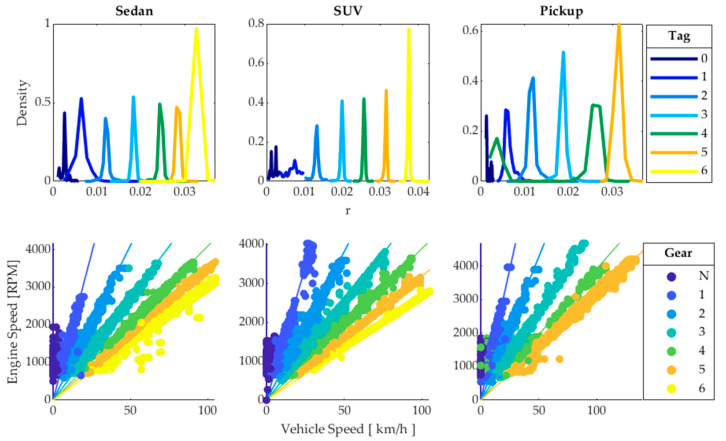
Obtaining labels through K-means and CT training.

The test vehicles are equipped with manual transmission, and like 69% of the vehicles sold in Ecuador [21], they do not have sensors to determine the gear selected by the driver; therefore, it is necessary to determine this information from the OBD data using machine learning according to the process shown in [17]. The K-means algorithm is applied to the data acquired in the RDE test to cluster the vector *r*, which is calculated using Equation (12).

(12) $$r_{i}=\frac{{VSS}_{i}}{{RPM}_{i}}$$

where VSS is the vehicle speed and RPM is the engine speed obtained from the OBD. The algorithm generates a label for each of the 7, 7, and 6 groups obtained from their centroids [26]. The generated groups correspond to each of the 6, 6, and 5 gears plus the neutral position of the sedan, SUV, and pickup vehicles, respectively. With the obtained label, a classification tree (CT) is trained that is applicable to all sampled driving cycles, as shown in Figure 6.

**Figure 7:**
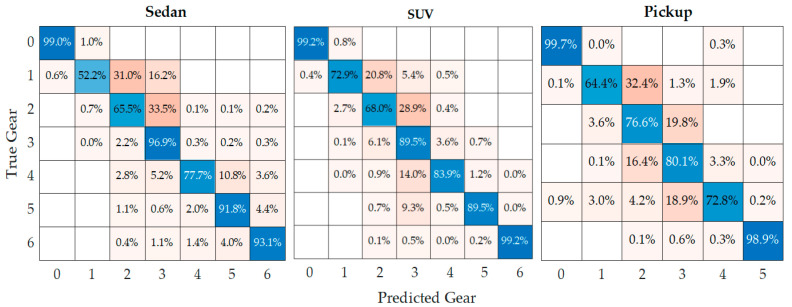
Confusion matrices.

The values of V_OBD_ and RPM are directly obtained through the OBD, so they cannot be used to train the GPS-based model. The gear used by the driver cannot be directly determined by V_GPS_ since the gears selected do not depend exclusively on the driving speed. Given that gear usage during driving is random [27], supervised learning is employed, where the forces acting on the vehicle’s movement are used as predictors for classification trees, and the gear used by the driver is the output, whose labels were obtained from OBD data, making the training vector *I* = [*V_GPS_, a_X_, F_r_, F_g_, F_a_*], [19]. From the training performed, three classification trees are obtained with 7, 7, and 6 splits to determine the gear of the sedan, SUV, and pickup vehicles, respectively; their training results are shown in the confusion matrices in Figure 7. These hyperparameters were determined based on the appropriate configuration of the maximum tree, which is quite simple, making pruning unnecessary. Cross-validation of the obtained trees is performed by randomly splitting the training data into several mutually exclusive folds. In each fold, a portion of the data is used for training and another portion for testing [28]. The data are divided into 5 folds, with each fold divided into 70% of the data for training and 30% for testing, resulting in an average test accuracy rate of 99.5%. The highest accuracy rates occur in neutral, 5th, and 6th gears, while in 3rd and 4th gears, the model’s efficiency decreases because the vehicle’s performance under these conditions is very similar.

### 2.6. Estimation of the Relative Importance of Each Predictor

Predictive models based on machine learning methods such as random forest (RF) suffer from bias and variance issues. Simple models have low variance and high bias, whereas complex models reduce bias but increased variance due to overfitting [29]. Therefore, the training process of ANNs is optimized by prioritizing the use of the most important predictors determined by the RF technique [30], which coincides with the selection according to the Gini criterion. RF relies on multiple classification and regression trees (CART) to mitigate dimensionality problems in predicting variables, thereby enhancing the accuracy and stability of the model obtained by averaging the results of individual CART models [31]. This approach is applied to datasets where not all variables are considered, as they are randomly chosen in each CART [32].

**Figure 8:**
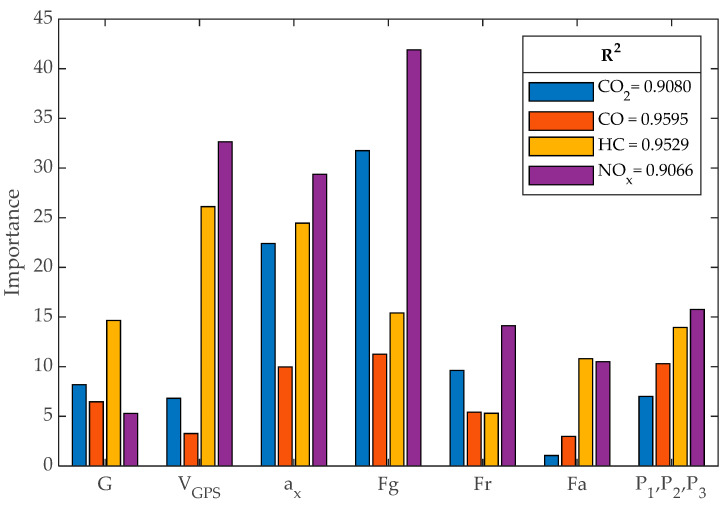
Predictor importance.

For variable selection with RF, the data obtained from the RDE of Route 1 for each test vehicle were considered. The inputs included all vehicle operating parameters obtained through GPS, while the outputs consisted of the resulting pollutant emissions. To reduce the variance contributed by the predictors to the model, a very effective technique called “bagging” was employed. This involves combining results from different CARTs obtained using different subsets of predictors from the same population [31]. For this purpose, continuous variables must be transformed into categorical variables through level discrimination [17]. The number of levels was set to 7, 110, 144, 144, 144, 144, 3, 3, and 3 for the variables *G*, *V_GPS_*, *a_x_*, *F_g_*, *F_r_*, *F_a_*, *P_1_*, *P_2_*, and *P_3_*, respectively. The outcome of the most influential predictors is illustrated in Figure 8. The R^2^ factor estimates the quality of the fit that RF has achieved to determine the importance of the variables in each of the outputs [33]. It is determined by Equation (13), where $Y_{i}$ is the vector of n predictions, ${\hat{Y}}_{i}$ is the vector of true values, and ${\bar{Y}}_{i}$ is their mean value.

(13) $$R^{2}=\frac{\sum_{i=1}^{n}{\left(Y_{i}-{\hat{Y}}_{i}\right)}^{2}}{\sum_{i=1}^{n}{\left(Y_{i}-{\bar{Y}}_{i}\right)}^{2}}$$

### 2.7. Training of the Neural Network with the Most Significant Variables

The data obtained on Route 1 of the RDE test for each vehicle were used to train 1 ANN for each pollutant, with their respective input vectors being as follows:

*I_CO2_* = [*F_g_*, *a_X_*, *G*, *V*_*GPS*_] (14)

*I_CO_* = [*F_g_*, *a_X_*, *G*, *V_GPS_*, *P*] (15)

*I*_*NOX*_ = [*V_GPS_*, *a_X_*, *F_g_*, *G*] (16)

*I*_*HC*_ = [*F_g_*, *V_GPS_*, *a_X_*, *P*] (17)

The networks were configured with 4 neurons in the input layer, 10 in the hidden layer, and 1 in the output layer, as determined in [34]. The dataset from Route 1 was divided into 70% for training, 15% for validation, and the remaining 15% for testing. The Levenberg–Marquardt backpropagation algorithm was used for network training, employing backpropagation to increase the learning speed [35,36]. The training characteristics of the ANNs obtained for estimating CO_2_, CO, NO_X_, and HC are shown in Table 5, where it can be observed that generalization is achieved rapidly, avoiding network overfitting. This can be verified by comparing the cost values (mean squared error, MSE) in training, validation, and testing, where the indicator’s value in the test dataset is lower than in training. The MSE is calculated using Equation (18), where $Y_{i}$ is the vector of *n* predictions and ${\hat{Y}}_{i}$ is the vector of true values [33].

(18) $$MSE=\frac{1}{n}\sum_{i=1}^{n}{{(Y}_{i}-{\hat{Y}}_{i})}^{2}$$

The networks for estimating CO_2_, CO, NO_X_, and HC were trained achieved in 221, 344, 101, and 17 epochs, respectively, due to early stopping, ensuring good performance of the networks in the training, validation, and testing stages. The number of epochs is relatively low for estimating HC, as generalization is quickly reached, avoiding network overfitting. This can be verified by comparing the MSE values.

### 2.8. Validation of the Neural Networks

**Figure 9:**
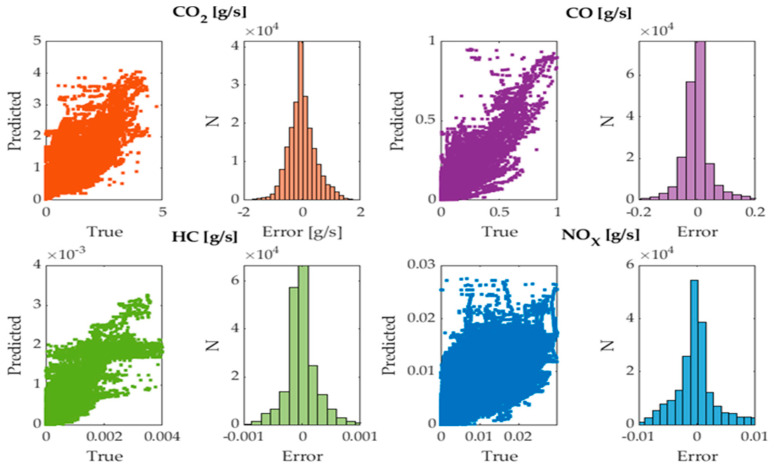
ANN errors.

The obtained networks were applied using the data collected on Route 2 of the RDE test for the three vehicles as inputs to compare the results with the data measured by the PEMS. It was observed that the fit is very satisfactory according to the scatter plots and error distribution diagrams shown in Figure 9. The model errors exhibit a nearly normal symmetric behavior around 0, with no offsets in the estimation of each contaminant [37]. Moreover, they behave completely randomly, thus ruling out the inference of other variables not considered in the ANNs’ training.

## 3. Results

To assess the performance of the parametric model based on GPS for emission estimation, its results are compared to those obtained by applying the IVE model and the OBD-based estimation model [17].

### 3.1. CO_2_ Emissions

**Figure 10:**
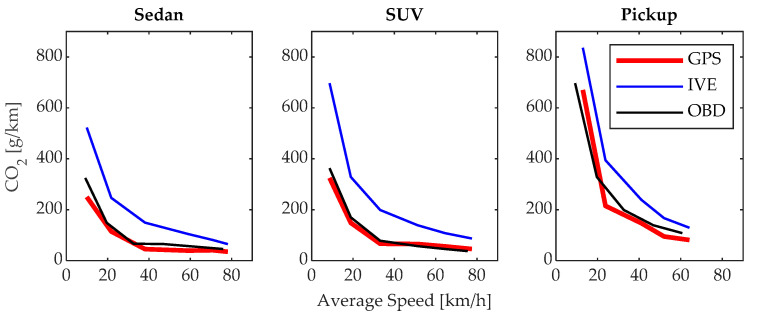
CO_2_ emissions.

The emission of CO_2_ depends on the average driving speed. In Figure 10, the results obtained for the three analyzed vehicles are shown; in all three cases, the CO_2_ emissions are inversely proportional to the average driving speed, and the results of the IVE model are higher than those obtained by the other models, in accordance with what was shown in Section 1 [5]. The highest emissions are 249.91, 324.55, and 670.61 g/km, achieved at 9.95, 8.65, and 12.95 km/h using the first gear, while the lowest emissions are 35.1, 45.58, and 80.98 g/km, achieved at 78.15, 77.48, and 64.15 km/h using the highest gear in the sedan, SUV, and pickup vehicles, respectively. If a comparison is made among the three test vehicles, it can be observed that the highest emission values are found in the pickup, followed by the SUV and sedan; these values are proportional according to their weight and aerodynamics, among other factors. It is worth noting the close similarity between the results generated by the proposed model and the OBD-based model, as both are based on a large amount of data collected under real driving conditions.

### 3.2. CO Emissions

**Figure 11:**
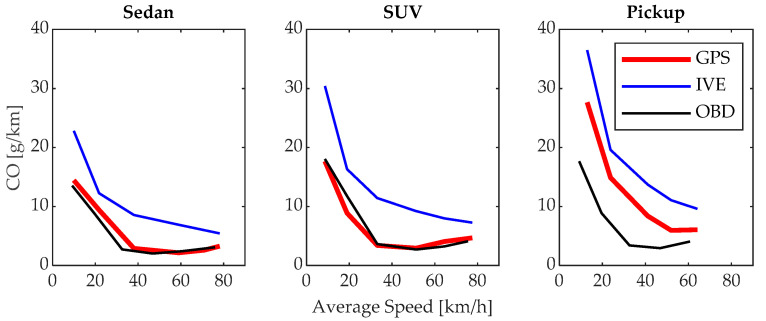
CO emissions.

The emissions of CO shown in Figure 11 are inversely proportional to the average driving speed. The maximum emissions values are 18.04, 17.65, and 27.65 g/km, achieved when driving at the minimum average speed using first gear. As the driving speed increases, the minimum CO emissions are achieved, with values of 2.70, 2.95, and 5.95 g/km when using the fourth gear in the sedan, SUV, and pickup vehicles, respectively. When using gears higher than fourth gear, the emissions slightly increase, highlighting the importance of efficient driving and proper gear usage to reduce pollutant emissions. For the sedan and SUV vehicles, the results of the proposed model and the OBD-based model are very similar. However, there is a difference in the results for the pickup vehicle; this is because these vehicles are used as light-duty vehicles [38], which increases the engine load and consequently CO emissions due to incomplete fuel combustion [39].

### 3.3. HC Emissions

**Figure 12:**
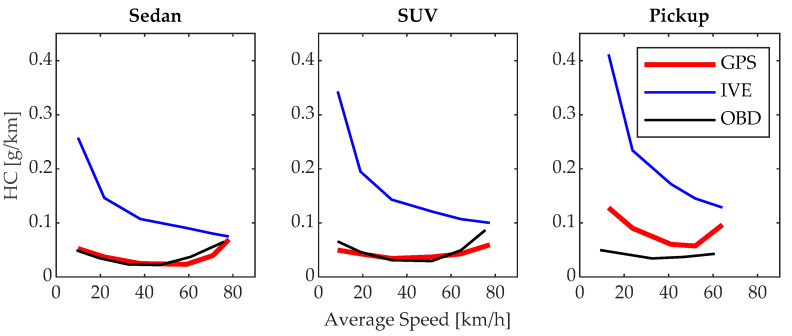
HC emissions.

The HC emissions determined by the proposed model are very similar to those estimated by the OBD model in the sedan and SUV vehicles, with differences observed in the pickup category, as explained in Section 3.2. As shown in Figure 12, in all three vehicles, the emission factor is high at low speed values and high driving speeds, reaching a minimum emissions value of 0.0235, 0.0343, and 0.0573 g/km at 58.98, 51.28, and 51.98 km/h for the sedan, SUV, and pickup vehicles, respectively. Beyond this speed, HC emissions increase again. This occurs because at low speeds, the loading and RPM conditions are not optimal for generating efficient and complete combustion, while at high speeds, the loading and temperature conditions also affect combustion efficiency [39]. However, the behaviors are similar in all three test vehicles, indicating that the model is effective.

### 3.4. NO_X_ Emissions

**Figure 13:**
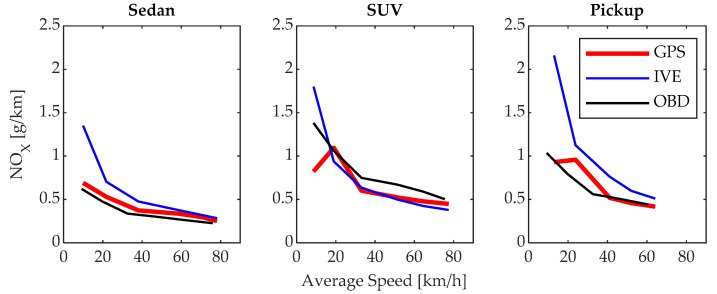
NO_X_ Emissions.

The emissions of NOx represented in Figure 13 show that the proposed model and the IVE model maintain the same behavior in the sedan vehicle, with the maximum emissions being 0.6907 g/km in first gear at a speed of 9.95 km/h. After this point, the NOx emissions decrease as the average driving speed increases because, at lower speeds, the engine tends to experience a higher load, which is a crucial factor for NO_x_ emissions. In the SUV and pickup vehicles, the maximum emissions of 1.094 and 0.958 g/km occur in second gear at an average speed of 18.94 and 23.79 km/h, respectively; this is because this gear is used to gain speed after starting, resulting in an increase in temperature and pressure in the combustion chamber in light-duty vehicles [23,40]. This demonstrates that the proposed model is capable of replicating results from a reference model, thus supporting its effectiveness and validity.

## 4. Discussion

To evaluate the performance of the proposed model, its results are compared with those obtained using the RDE test. The average emission factors for each model, determined from the total pollutant emissions and total distance traveled, are shown in Table 6. It is noteworthy that there is a close resemblance between the results of the RDE and GPS models; small differences arise because the relationship between the urban, rural, and highway segments in the RDE driving cycle differs from what occurs during random driving, which provided the data used for the GPS model estimation, whereas the values estimated by the IVE model are higher than the other models analyzed, as indicated in Section 1 [5]. The main difference lies in the CO_2_ emissions factor, which, as already discussed, is strongly influenced by low driving speeds in urban areas. The emissions estimated by the proposed model show minimal deviations from the RDE results, with −3.97% in CO emission for the sedan vehicle and −15.56% in HC emissions and −3.57% in NO_X_ emissions for the SUV vehicle. The largest deviations occur in the estimation of emissions for the pickup vehicle due to the specific use of these types of vehicles [38]. Table 6 shows the average emission factor values for the three models analyzed.

The emissions of CO_2_, CO, and NOX exhibit similar behavior concerning speed, and this is attributed to the gear shifts of the vehicle according to the driving speed. At lower speeds, lower gears (first, second, and third) are engaged, requiring the engine to operate at higher speeds, thereby increasing air and fuel consumption and, consequently, emissions. Conversely, at higher speeds, higher gears (fourth, fifth, and sixth) are utilized, reducing the engine’s rotation speed and thus fuel consumption and emissions generated [17].

## 5. Conclusions

This article proposes a novel approach for estimating pollutant emissions from the most representative light vehicles circulating in Ecuador based on GPS data and applying machine learning to a large dataset. An approach was developed that initially employs a highly effective classifier to assess the gears selected by the driver. This classifier was built by obtaining labels through K-means clustering and subsequent training of classification trees. Errors manifest in the brief intervals that occur during gear transitions. Pollutant emissions calculations were performed by determining the importance of predictors in the data collected from two RDE test routes using RF. Subsequently, four ANNs were trained, which demonstrated high determination coefficients R^2^ of 0.735, 0.861, 0.892, and 0.798 for the estimation of CO_2_, CO, HC, and NO_X_, respectively, and adequate error behavior, validating the method used.

In urban environments, average driving speeds are reduced, leading to the predominant use of the first, second, and third gears, resulting in a consequent increase in pollutant emission factors. In this context, the proposed model demonstrates greater robustness to various traffic conditions and driving styles in urban areas. This is because the model is based on the results of random driving data covering 324, 300, and 316 km compared to the 96.99, 81.88, and 87.21 km of the RDE test and the results of the IVE model for sedan, SUV, and pickup vehicles, respectively. As the average driving speed increases, the results of the proposed model and the RDE test become more similar due to the decreased influence of traffic on vehicle performance and the smaller number of transient events in driving.

The obtained model is characterized by estimating emissions at a microscopic level with high reliability and low cost, due to the current availability of GPS receivers in a variety of portable devices. It presents advantages over existing models such as the IVE model, as it considers traffic conditions, the physical states of roads, and all interactions and dynamics between vehicles and their surroundings. Additionally, it considers special environmental conditions such as mountainous terrain and altitude above sea level, as well as the specific environmental conditions of each region, such as temperature, humidity, atmospheric pressure, and solar radiation.

The obtained model offers economic and practical advantages in its application compared to other models, given the ease of generating applications for installation on portable devices. Furthermore, it shows highly satisfactory performance, as despite its limitations, it provides excellent results in pollutant estimation without the need for connection to expensive equipment for long periods of time. This work presents several limitations such as vehicle longevity, driving styles, cold operation, and circulation on slopes, as under these operating conditions, engine control systems tend to employ special strategies that directly influence emission behavior, so they should be considered for future developments. It is essential to replicate the proposed methodology in models of vehicles with a greater presence and activity in the automotive fleet, aiming to refine the results of vehicle emissions inventories.

## Figures and Tables

**Table 1:** Characteristics of the test vehicles.

Vehicle	Type	Displacement ([cc)]	Odometer (km)	Weight (kg)	*Cx*	*A_f_* (m^2^)
A	Sedan	1400	28,678	1580	0.32	1.83
B	SUV	2000	18,720	1719	0.33	3.015
C	Pickup	2400	43,657	2745	0.39	3.201

**Table 2:** Driving data obtained.

Parameter	Symbol	Source	Unit
Latitude	Lat	GPS	(°)
Longitude	Lon	GPS	(°)
Altitude	Alt	GPS	(m.a.s.l.)
Vehicle Speed	V_GPS_	GPS	(km/h)
Vehicle Speed	V_OBD_	OBD	(km/h)
Engine Speed	RPM	OBD	(RPM)
Acceleration	a_X_	Calculated	(m/s^2^)
Fuel Flow	$\dot{m_{f}}$	AIC 5004	(L/h)
Carbon dioxide	CO_2_	AGS-688	(%)
Carbon monoxide	CO	AGS-688	(%)
Nitrous oxides	NO_X_	AGS-688	(ppm)
Hydrocarbons	HC	AGS-688	(ppm)

**Table 3:** Characteristics of the RDE tests.

RDE Trip Characteristics	SUV	Sedan	Pickup	RDE Trip Requirements	Unit
Sample number	85,697	55,915	60,325	-	-
Total distance	62.49	58.39	47.75	-	(km)
Total duration	96.99	81.88	87.21	90–120	(min)
Urban distance	21.63	15.89	16.32	>16	(km)
Rural distance	21.24	24.51	15.37	>16	(km)
Motorway distance	19.61	17.98	16.06	>16	(km)
Urban distance share	34.61	27.22	34.26	29–44	(%)
Rural distance share	34.01	41.98	31.76	23–43	(%)
Motorway distance share	31.38	30.78	36.06	23–43	(%)
Urban average speed	22.49	22.10	16.02	-	(km/h)
Rural average speed	50.14	64.02	53.02	-	(km/h)
Motorway average speed	85.19	68.43	102.46	-	(km/h)
Urban center time	11.61	8.41	29.46	10–30	(%)
Altitude difference	−4.4	21.3	25.8	<100	(m)

**Table 4:** Average emission factors in RDE.

F	Sedan ([g/km)]	SUV ([g/km)]	Pickup ([g/km)]
CO_2_	54.72	70.23	102.13
CO	5.28	5.33	9.74
HC	0.0374	0.0485	0.0656
NO_X_	0.3527	0.7199	0.616

**Table 5:** Training characteristics.

	CO_2_		CO		HC		NO_X_	
	R	MSE	R	MSE	R	MSE	R	MSE
Training	0.7344	0.2158	0.8566	0.0033	0.8924	8.155 × 10^−8^	0.7983	1.658 × 10^−5^
Validation	0.7353	0.2165	0.8536	0.0034	0.8944	7.821 × 10^−8^	0.7963	1.652 × 10^−5^
Test	0.7358	0.2183	0.8616	0.0032	0.8920	7.974 × 10^−8^	0.7992	1.647 × 10^−5^

**Table 6:** Average emission factors.

F	Sedan ([g/km)]			SUV ([g/km)]			Pickup ([g/km)]		
	IVE	RDE	GPS	IVE	RDE	GPS	IVE	RDE	GPS
CO_2_	157.18	54.72	60.97	208.97	70.23	80.93	243.61	102.13	108.66
CO	9.46	5.28	5.07	11.83	5.33	6.95	14.19	9.74	12.68
HC	0.112	0.0374	0.0299	0.1477	0.0485	0.041	0.168	0.0656	0.0912
NO_X_	0.531	0.3527	0.403	0.661	0.7199	0.694	0.792	0.616	0.762

## Data Availability

Data are contained within the article.

## Abbreviations

Abbreviation	Variable
α	Hydrogen molar ratio
*A_f_*	Frontal area of the vehicle
Alt	Altitude
ANN	Artificial neural network
*a_x_*	Longitudinal acceleration
CART	Classification and regression trees
*C_dry_*	Dry basis emissions
CO	Carbon monoxide
CO_2_	Carbon dioxide
CSV	Comma separated values
CT	Classification tree
*C_wet_*	Wet basis emission
*C_X_*	Drag coefficient
*EF*	Emission factor
*F*	Coefficients of static adherence
*f*_0_	Coefficients of dynamic adherence
*F_a_*	Aerodynamic resistance
*F_g_*	Gravitational resistance
*F_r_*	Rolling resistance force
GPS	Global position system
HC	Hydrocarbons
IVE	International vehicle emissions
*I_CO2_*	*CO_2_* Input vector
*I_CO_*	*CO* Input vector
*I_HC_*	HC Input vector
*I_NOX_*	NO_X_ Input vector
*Kw*	correction factor
Lat	Latitude
Lon	Longitude
*ṁ*	Instantaneous mass emissions
*ṁ_e_*	Exhaust mass flow
*ṁ_f_*	Fuel flow
*ṁ_i_*	Air mass flow
MSE	Mean squared error
NO_X_	Nitrous oxides
OBD	On-board diagnostics
PEMS	Portable emissions measurement system
RDE	Real driving emissions
*p*	Air density
RF	Random forest
SUV	Sports utility vehicle
V_OBD_	Vehicle speed obtained from OBD
V_GPS_	Vehicle speed obtained from GPS
*µ*	Ratio between the density of each component and the exhaust

## References

[1] Smit R., Kingston P., Wainwright D., Tooker R. (2017). A tunnel study to validate motor vehicle emission prediction software in Australia. Atmos. Environ..

[2] Bond T.C., Scott C.E. (2022). Aerosol and precursor gas emissions. Aerosols and Climate.

[3] Deng F., Lv Z., Qi L., Wang X., Shi M., Liu H. (2020). A big data approach to improving the vehicle emission inventory in China. Nat. Commun..

[4] Li P., Lu Y., Wang J. (2020). The effects of fuel standards on air pollution: Evidence from China. J. Dev. Econ..

[5] Mangones S.C., Jaramillo P., Fischbeck P., Rojas N.Y. (2019). Development of a high-resolution traffic emission model: Lessons and key insights from the case of Bogotá, Colombia. Environ. Pollut..

[6] Ortenzi F., Costagliola M.A. (2010). A New Method to Calculate Instantaneous Vehicle Emissions Using OBD Data.

[7] Costagliola M.A., Costabile M., Prati M.V. (2018). Impact of road grade on real driving emissions from two Euro 5 diesel vehicles. Appl. Energy.

[8] Kurtyka K., Pielecha J. (2019). The evaluation of exhaust emission in RDE tests including dynamic driving conditions. Transp. Res. Procedia.

[9] Mera Z., Fonseca N., López J.-M., Casanova J. (2019). Analysis of the high instantaneous NOx emissions from Euro 6 diesel passenger cars under real driving conditions. Appl. Energy.

[10] Fontaras G., Zacharof N.-G., Ciuffo B. (2017). Fuel consumption and CO_2_ emissions from passenger cars in Europe—Laboratory versus real-world emissions. Prog. Energy Combust. Sci..

[11] Samaras C., Tsokolis D., Toffolo S., Magra G., Ntziachristos L., Samaras Z. (2019). Enhancing average speed emission models to account for congestion impacts in traffic network link-based simulations. Transp. Res. Part D Transp. Environ..

[12] Prakash S., Bodisco T.A. (2019). An investigation into the effect of road gradient and driving style on NOX emissions from a diesel vehicle driven on urban roads. Transp. Res. Part D Transp. Environ..

[13] Boulter P.G., Barlow T.J., Mccrae I.S., Latham S., Parkin C. (2009). Emission Factors 2009: Report 1—A Review of Methods for Determining Hot Exhaust Emission Factors for Road Vehicles.

[14] Eckert J.J., Santiciolli F.M., Yamashita R.Y., Corrêa F.C., Silva L.C., Dedini F.G. (2019). Fuzzy gear shifting control optimisation to improve vehicle performance, fuel consumption and engine emissions. IET Control Theory Appl..

[15] Eckert J.J., Santiciolli F.M., Bertoti E., Costa E.D.S., Corrêa F.C., Silva L.C.D.A.E., Dedini F.G. (2018). Gear shifting multi-objective optimization to improve vehicle performance, fuel consumption, and engine emissions. Mech. Based Des. Struct. Mach..

[16] Larue G.S., Malik H., Rakotonirainy A., Demmel S. (2014). Fuel consumption and gas emissions of an automatic transmission vehicle following simple eco-driving instructions on urban roads. IET Intell. Transp. Syst..

[17] Rivera-Campoverde N.D., Muñoz-Sanz J.L., Arenas-Ramirez B.d.V. (2021). Estimation of pollutant emissions in real driving conditions based on data from OBD and machine learning. Sensors.

[18] Paredes R., Cardoso J.S., Pardo X.M. (2015). Pattern Recognition and Image Analysis: 7th Iberian Conference, IbPRIA 2015 Santiago de Compostela, Spain, 17–19 June 2015.

[19] Rivera-Campoverde N., Sanz J.M., Arenas-Ramirez B. (2023). Low-Cost Model for the Estimation of Pollutant Emissions Based on GPS and Machine Learning. Proceedings of the XV Ibero-American Congress of Mechanical Engineering.

[20] Consejo de la Unión Europea, Reglamento de la Comisión Europea (2016). Por el que se Modifica el Reglamento (CE) n.o 692/2008 en lo que Concierne a las Emisiones Procedentes de Turismos y Vehículos Comerciales Ligeros (Euro 6).

[21] Asociación de Empresas Automotrices del Ecuador Automotive Sector in Figures; Quito, Ecuador, 2023. https://www.aeade.net/boletin-sector-automotor-en-cifras/.

[22] Wen M., Zhang C., Yue Z., Liu X., Yang Y., Dong F., Liu H., Yao M. (2020). Effects of Gasoline Octane Number on Fuel Consumption and Emissions in Two Vehicles Equipped with GDI and PFI Spark-Ignition Engine. J. Energy Eng..

[23] Campoverde P.A.M., Campoverde N.D.R., Espinoza J.E.M., Fernandez G.M.R., Novillo G.P. (2022). Influence of the road slope on NOx emissions during start up. Mater. Today Proc..

[24] Frank T., Turney J. (2016). Aerodynamics of commercial vehicles. Lecture Notes in Applied and Computational Mechanics.

[25] Kourta A., Gilliéron P. (2009). Impact of the Automotive Aerodynamic Control on the Economic Issues. www.iafiTionline.net.

[26] Jain A.K. (2010). Data clustering: 50 years beyond K-means. Pattern Recognit. Lett..

[27] Yasami Y., Pour Mozaffari S. (2010). A novel unsupervised classification approach for network anomaly detection by k-Means clustering and ID3 decision tree learning methods. J. Supercomput..

[28] De’ath G., Fabricius K.E. (2000). Classification and regression trees: A powerful yet simple technique for ecological data analysis. Ecology.

[29] Archer K.J., Kimes R.V. (2008). Empirical characterization of random forest variable importance measures. Comput. Stat. Data Anal..

[30] Visser L., AlSkaif T., van Sark W. The Importance of Predictor Variables and Feature Selection in Day-ahead Electricity Price Forecasting. Proceedings of the 2020 International Conference on Smart Energy Systems and Technologies (SEST).

[31] Liang G., Zhu X., Zhang C. (2011). An Empirical Study of Bagging Predictors for Different Learning Algorithms. www.aaai.org.

[32] Probst P., Wright M.N., Boulesteix A. (2019). Hyperparameters and tuning strategies for random forest. Wiley Interdiscip. Rev. Data Min. Knowl. Discov..

[33] Karijadi I., Chou S.-Y. (2022). A hybrid RF-LSTM based on CEEMDAN for improving the accuracy of building energy consumption prediction. Energy Build..

[34] Kanellopoulos I., Wilkinson G.G. (1997). Strategies and best practice for neural network image classification. Int. J. Remote Sens..

[35] Sapna S. (2012). Backpropagation Learning Algorithm Based on Levenberg Marquardt Algorithm.

[36] Reynaldi A., Lukas S., Margaretha H. Backpropagation and Levenberg-Marquardt algorithm for training finite element neural network. Proceedings of the UKSim-AMSS 6th European Modelling Symposium, EMS 2012.

[37] Shanmugam B.K., Vardhan H., Raj M.G., Kaza M., Sah R., Hanumanthappa H. (2021). ANN modeling and residual analysis on screening efficiency of coal in vibrating screen. Int. J. Coal Prep. Util..

[38] Woody M., Vaishnav P., Keoleian G.A., De Kleine R., Kim H.C., Anderson J.E., Wallington T.J. (2022). The role of pickup truck electrification in the decarbonization of light-duty vehicles. Environ. Res. Lett..

[39] Kean A.J., Harley R.A., Kendall G.R. (2003). Effects of vehicle speed and engine load on motor vehicle emissions. Environ. Sci. Technol..

[40] O’Driscoll R., ApSimon H.M., Oxley T., Molden N., Stettler M.E., Thiyagarajah A. (2016). A Portable Emissions Measurement System (PEMS) study of NOx and primary NO2 emissions from Euro 6 diesel passenger cars and comparison with COPERT emission factors. Atmos. Environ..

